# Supplementary material for: Germline molecular data in hereditary breast cancer in Brazil: Lessons from a large single-center analysis
Source: PLoS One. 2021 Feb 19;16(2):e0247363. doi: 10.1371/journal.pone.0247363 (PMC7895369; doi:10.1371/journal.pone.0247363)
Supplement: S1 Table — (DOCX) [file pone.0247363.s001.docx]

**S1 Table: Characteristics of genetic tests performed.**

| Laboratory | Number of patients | Panel with CNV analysis | Number of genes* | Coverage | Variant Classification Framework |
| --- | --- | --- | --- | --- | --- |
| Invitae | 196 | S | 80-84 | 50 reads | ACMG/AMP**+ Sherloc*** |
| Fleury | 13 | S | 138 | 50 reads | ACMG/AMP |
| Mendelics | 5 | S | 37 | 10 reads | ACMG/AMP |
| Color | 2 | S | 30 | 20 reads | ACMG/AMP |
| Santa Paula | 3 | N/A | 16 | 10 reads | ACMG/AMP |
| Total | 219 |  |  |  |  |

Abbreviations: CNV, Copy Number Variant; ACMG, American College of Medical Genetics; AMP, Association for Molecular Pathology.

* The following genes were included in all the genetic tests: BRCA1, BRCA2, BRIP1, MLH1, MSH2, MSH6, PMS2, RAD51C, RAD51D, STK11, ATM, CDH1, CHEK2, PALB2, PTEN, TP53.

** Methodology described in Richards et al., 2015.

*** Methology described in Nykamp et al., 2017.
