# Supplementary material for: Germline molecular data in hereditary breast cancer in Brazil: Lessons from a large single-center analysis
Source: PLoS One. 2021 Feb 19;16(2):e0247363. doi: 10.1371/journal.pone.0247363 (PMC7895369; doi:10.1371/journal.pone.0247363)
Supplement: S2 Table — (DOCX) [file pone.0247363.s002.docx]

**S2 Table: Clinical characteristics of patients with pathogenic and likely pathogenic variants.**

| **Identification** | **Gene** | **Pathogenic Variant** | **Other Pathogenic Variants** | **Gender** | **Age at diagnosis of breast cancer** | **Number of primary tumors** | **Other tumors** | **Laterality** | **Histology** | **IHC** | **Tumor Grade** | **Family History of cancer** | **Fulfill NCCN 2020 criteria** |
| --- | --- | --- | --- | --- | --- | --- | --- | --- | --- | --- | --- | --- | --- |
| P050 | *ATM* | c.7913G>A (p.Trp2638*) | No | F | 35 | 1 | No | Unilateral | NTS | RE+/ RP+/ HER2- | 3 | Positive | Yes |
| P027 | *BARD1* | c.176_177del (p.Glu59Alafs*8) | No | F | 53 | 1 | No | Unilateral | NTS | RE+/ RP+/HER2+ | 1 | Positive | Yes |
| P101 | *BARD1* | c.176_177del (p.Glu59Alafs*8) | No | F | 57 | 2 | No | Metachronic  bilateral | NTS | RE-/ RP-/HER2+ | N/A | Positive | Yes |
| P242 | *BRCA1* | c.132C>G (p.Cys44Trp) | No | F | 40 | 1 | No | Unilateral | NTS | Triple negative | 3 | Negative | Yes |
| P202 | *BRCA1* | c.441+2T>A (splice donor) | No | F | 50 | 1 | Thyroid, renal, parotid | Unilateral | NTS | Triple negative | N/A | Positive | Yes |
| P181 | *BRCA1* | c.791_794del (p.Ser264Metfs*33) | No | F | 63 | 1 | No | Unilateral | NTS | RE+/ RP+/ HER2- | 2 | Positive | No |
| P037 | *BRCA1* | c.850C>T (p.Gln284*) | No | F | 34 | 1 | Thyroid | Unilateral | NTS | RE+/ RP-/ HER2 - | 3 | Negative | Yes |
| P029 | *BRCA1* | c.1115G>A (p.Trp372*) | No | F | 22 | 1 | No | Unilateral | NTS | N/A | 3 | Positive | Yes |
| P053 | *BRCA1* | c.1687C>T (p.Gln563*) | No | M | 78 | 1 | Breast | Unilateral | NTS | RE+/ RP+/ HER2- | 3 | N/A | Yes |
| P063 | *BRCA1* | c.1687C>T (p.Gln563*) | No | F | 40 | 1 | No | Unilateral | NTS | RE+/ RP+/HER2+ | N/A | Positive | Yes |
| P207 | *BRCA1* | c.3598C>T p.Gln1200* | No | F | 31 | 1 | No | Unilateral | DCIS | N/A | 3 | Positive | Yes |
| P110 | *BRCA1* | c.5177_5180delGAAA (p.Arg1726Lysfs*3) | No | F | 38 | 1 | No | Unilateral | NTS | Triple negative | 3 | Positive | Yes |
| P126 | *BRCA1* | c.5266dupC (p.Gln1756Profs*74) | No | F | 38 | 1 | No | Unilateral | NTS | RE+/ RP-/ HER2- | 3 | Positive | Yes |
| P247 | *BRCA1* | c.5266dupC  (p.Gln1756Profs*74) | No | F | 51 | 1 | No | Unilateral | NTS | Triple negative | 3 | Positive | Yes |
| P002 | *BRCA1* | c.5266dupC (p.Gln1756Profs*74) | No | F | 42 | 1 | No | Unilateral | NTS | Triple negative | 3 | Positive | Yes |
| P127 | *BRCA1* | del exons 8-19 | CTC1 c.2346delG (p.Leu783Cysfs*38) | M | 26 | 1 | No | Unilateral | NTS | RE+/ RP+/ HER2+ | 2 | Positive | Yes |
| P091 | *BRCA2* | c.156_157insAlu (p.Lys53Alafs) | No | F | 34 | 1 | Head and neck | Unilateral | NTS | RE+/ RP+/ HER2- | 2 | Positive | Yes |
| P070 | *BRCA2* | c.1310_1313delAAGA (p.Lys437Ilefs*22 | MUTYH c.536 A>G (p.Tyr179Cys) | F | 56 | 2 | No | Synchronous bilateral | NTS/ NTS | RE+/ RP+/ HER2- | N/A | Positive | Yes |
| P105 | *BRCA2* | c.2512A>T (p.Lys838*) | No | M | 72 | 1 | Breast | Unilateral | N/A | N/A | N/A | Positive | Yes |
| P006 | *BRCA2* | c.3680_3681del (p.Leu1227Glnfs*5) | No | F | 63 | 1 | No | Unilateral | NTS | Triple negative | 3 | Positive | Yes |
| P178 | *BRCA2* | c.5073dupA (p.Trp1692Metfs*3) | No | F | 64 | 1 | No | Unilateral | NTS | RE+/ RP-/ HER2- | 3 | Positive | Yes |
| P021 | *BRCA2* | c.6405_6409del (p.Asn2135Lysfs*3) | No | F | 42 | 2 | Breast | Synchronous bilateral | NTS/ ILC | RE+/ RP+/ HER2 - | 2/2 | Positive | Yes |
| P167 | *CHEK2* | c.319+2T>A (splice donor) | No | F | 44 | 1 | No | Unilateral | DCIS | N/A | N/A | Positive | Yes |
| P031 | *CHEK2* | c.349A>G (p.Arg117Gly) | No | F | 38 | 3 | Thyroid | Metachronic  bilateral | NTS | RE+/ RP+/ HER2 - | N/A | Positive | Yes |
| P013 | *CHEK2* | c.478A>G (p.Arg160Gly) | No | F | 28 | 1 | No | Unilateral | NTS | RE+/RP+/ HER2 N/A | N/A | Positive | Yes |
| P097 | *CHEK2* | c.593-1G>T (splice acceptor) | No | F | 52 | 1 | No | Unilateral | NTS | RE+/ RP+/ HER2- | 3 | Positive | Yes |
| P183 | *CHEK2* | c.846+1G>C (splice donor) | No | F | N/A | 4 | No | Unilateral | NTS | RE+/ RP+/ HER2 N/A | 1 | Positive | Yes |
| P023 | *CHEK2* | c.1008+2T>G (splice donor) | No | F | 45 | 2 | Breast | Metachronic  bilateral | NTS /DCIS | RE+/ RP+/HER2+ | 1/3 | Positive | Yes |
| P248 | *PALB2* | deletion exon 2-3 | No | F | 39 | 1 | No | Unilateral | NTS | RE-/ RP-/ HER2+ | 3 | Positive | Yes |
| P004 | *RAD51C* | c.709C>T (p.Arg237*) | No | F | 53 | 1 | No | Unilateral | NTS | RE+/ RP+/HER2+ | 2 | Positive | Yes |
| P223 | *RAD51C* | c.709C>T (p.Arg237*) | No | F | 51 | 2 | No | Synchronous bilateral | NTS | RE+/ RP+/ HER2- | 3/3 | Positive | Yes |
| P089 | *RAD51D* | c.694C>T (p.Arg232*) | No | F | 34 | 1 | No | Unilateral | NTS | RE+/ RP-/ HER2- | 3 | Positive | Yes |
| P121 | *RECQL4* | c.1166_1167del (p.Cys389Phefs*33) | No | F | 27 | 1 | Breast | Unilateral | N/A | RE+/ RP+/ HER2+ | N/A | Positive | Yes |
| P102 | *TP53* | c. 733G>A (p.Gly245Ser) | No | F | 20 | 1 | Thyroid | Unilateral | NTS | RE+/ RP+/ HER2- | N/A | Positive | Yes |
| P043 | *TP53* | c.1010G>A (p.R337H) | No | F | 42 | 2 | Breast | Unilateral | NTS | RE+/ RP+/ HER2 - | 1 | Positive | Yes |
| P052 | *TP53* | c.1010 G>A (p.Arg337His) | No | F | 39 | 1 | Gallbladder | Unilateral | NTS | N/A | 2 | Positive | Yes |
| P067 | *TP53* | c.1010A>G (p.His337Arg) | No | F | 59 | 2 | No | Unilateral | NTS | RE+/ RP-/HER2+ | 3 | Positive | Yes |
| P194 | *TP53* | c.1010G>A (p.Arg337His) | No | F | 44 | 1 | No | Unilateral | NTS | RE+/ RP+/ HER2 N/A | N/A | Positive | Yes |
| P155 | *TP53* | c.1010G>A (p.Arg337His) | No | F | 42 | 1 | No | Unilateral | NTS | RE+/ RP+/ HER2- | 2 | Positive | Yes |
| P169 | *TP53* | c.1010G>A (p.Arg337His) | No | F | 59 | 5 | No | Unilateral | N/A | N/A | N/A | Positive | Yes |
| P135 | *TP53* | Partial deletion exon 5 | No | F | 36 | 1 | No | Unilateral | N/A | RE+/ RP-/ HER2- | N/A | Positive | Yes |
| P193 | *MUTYH* | c.347-1G>C (p.?) | No | F | 53 | 1 | No | Unilateral | DCIS | RE+/ RP+/ HER2+ | 3 | Positive | Yes |
| P035 | *MUTYH* | c.933+3A>C | No | F | 33 | 2 | Breast | Unilateral | NTS | RE+/ RP-/ HER2 - | 3 | Positive | Yes |
| P095 | *MUTYH* | c.1187G>A (p.Gly396Asp) | No | F | 53 | 2 | Breast | Unilateral | ILC | N/A | N/A | Positive | Yes |
| P187 | *MUTYH* | c.1187G>A (p.Gly396Asp) | No | F | 28 | 1 | Schwannoma, leiomyosarcoma, melanoma, head and neck | Unilateral | NTS | RE+/ RP+/ HER2+ | N/A | Positive | Yes |
| P214 | *MSH6* | c.1519dupA (p.Arg507Lysfs*8) | No | F | 39 | 1 | No | Unilateral | NTS | RE+/ RP+/ HER2- | 3 | Positive | Yes |

Abbreviations: IHC, Immunohistochemistry; TN, Triple negative; ER, Estrogen receptor; PR, Progesterone receptor; HER2, Human Epidermal Growth Factor Receptor-type 2; NTS, Invasive carcinoma not specified; DCIS, ductal carcinoma *in situ*; ILC, invasive lobular carcinoma; N/A, not available; P, positive; N, negative; U, unilateral; B, bilateral; S, synchronous; M, metachronous.
