# Supplementary material for: Germline molecular data in hereditary breast cancer in Brazil: Lessons from a large single-center analysis
Source: PLoS One. 2021 Feb 19;16(2):e0247363. doi: 10.1371/journal.pone.0247363 (PMC7895369; doi:10.1371/journal.pone.0247363)
Supplement: S3 Table — (DOCX) [file pone.0247363.s003.docx]

**S3 Table: Number of patients who met current genetic testing criteria according to the genetic test result.**

|  | Meet NCCN criteria | Do not meet NCCN criteria | Total |
| --- | --- | --- | --- |
| Pathogenic/Likely pathogenic variants | 45 | 1 | 46 |
| Negative test/ VUS | 146 | 32 | 178 |
| Total | 191 | 33 | 224 |

Abbreviations: NCCN, National Comprehensive Cancer Network.
