# Supplementary material for: Germline molecular data in hereditary breast cancer in Brazil: Lessons from a large single-center analysis
Source: PLoS One. 2021 Feb 19;16(2):e0247363. doi: 10.1371/journal.pone.0247363 (PMC7895369; doi:10.1371/journal.pone.0247363)
Supplement: S4 Table — (DOCX) [file pone.0247363.s004.docx]

**S4 Table: Variants of Uncertain Significance detected.**

| Gene | Variant | Classification | [dbSNP](https://www.ncbi.nlm.nih.gov/snp/rs377349459) or Variation ID | Number of patients |
| --- | --- | --- | --- | --- |
| *AIP* | c.784G>A (p.Asp262Asn) | VUS | rs758918509 | 1 |
| *ALK* | c.234G>A (p.Ser78=) | VUS | rs1338599727 | 1 |
| *ALK* | c.1050G>A (p.Ser350=) | VUS | rs749145522 | 1 |
| *ALK* | c.2073C>G (p.Ser691Arg) | VUS | rs370435082 | 1 |
| *ALK* | c.3743G>A (p.Arg1248Gln) | VUS | rs368059424 | 1 |
| *ALK* | c.4186G>A (p.Ala1396Thr) | VUS | rs201768549 | 1 |
| *ALK* | c.4694A>G (p.Asn1565Ser) | VUS | rs1208409389 | 1 |
| *APC* | c.-145C>G (Non-coding) | VUS | rs986030414 | 1 |
| *APC* | c.170A>T (p.Asp57Val) | VUS | rs794729227 | 1 |
| *APC* | c.278T>A (p.Leu93His) | VUS | rs876658977 | 1 |
| *APC* | c.280C>G (p.Arg94Gly) | VUS | rs550945533 | 1 |
| *APC* | c.4124A>C (p.His1375Pro) | VUS | rs750884499 | 1 |
| *APC* | c.5392A>G (p.Asn1798Asp) | VUS | rs200794097 | 1 |
| *APC* | c.7225G>A (p.Gly2409Arg) | VUS | rs1554088147 | 1 |
| *APC* | c.7550A>G (p.Tyr2517Cys) | VUS | rs587783036 | 1 |
| *ATM* | c.749G>A (p.Arg250Gln) | VUS | rs56123940 | 1 |
| *ATM* | c.1595G>A (p.Cys532Tyr) | VUS | rs35963548 | 1 |
| *ATM* | c.2492A>G (p.Asp831Gly) | VUS | rs587781352 | 2 |
| *ATM* | c.2945G>A (p.Arg982His) | VUS | rs749471737 | 3 |
| *ATM* | c.2735A>G (p.Gln912Arg) | VUS | rs730881353 | 1 |
| *ATM* | c.3240C>A (p.Asp1080Glu) | VUS | rs149911447 | 1 |
| *ATM* | c.3630G>A (p.Met1210Ile) | VUS | rs587778073 | 1 |
| *ATM* | c.4414T>G (p.Leu1472Val) | VUS | rs539676759 | 1 |
| *ATM* | c.4775A>G (p.Glu1592Gly) | VUS | rs1565463405 | 1 |
| *ATM* | c.4916C>T (p.Pro1639Leu) | VUS | rs752459491 | 1 |
| *ATM* | c.5753G>C (p.Arg1918Thr) | VUS | rs148064985 | 1 |
| *ATM* | c.6543G>T (p.Glu2181Asp) | VUS | rs138828590 | 1 |
| *ATM* | c.7375C>G (p.Ag2459Gly) | VUS | rs730881383 | 1 |
| *ATM* | c.7475T>G (p.Leu2492Arg) | VUS | rs56399857 | 1 |
| *AXIN2* | c.727C>G (p.Leu243Val) | VUS | rs1060502152 | 1 |
| *AXIN2* | c.750G>T (p.Leu250Phe) | VUS | rs1060502149 | 1 |
| *AXIN2* | c.1387C>G (p.Arg463Gly) | VUS | rs730881395 | 1 |
| *AXIN2* | c.1701C>G (p.Ser567Arg) | VUS | Not reported | 1 |
| *BAP1* | c.1444T>G (p.Ser482Ala) | VUS | Variation ID: 947531 | 1 |
| *BAP1* | c.358A>G (p.Lys120Glu) | VUS | rs774573926 | 1 |
| *BAP1* | c.1058T>C (p.Ile353Thr) | VUS | rs754217069 | 1 |
| *BARD1* | c.841C>T (p.Pro281Ser) | VUS | rs200059956 | 1 |
| *BARD1* | c.2171C>T (p.Ala724Val) | VUS | rs587782662 | 1 |
| *BARD1* | c.2255A>C (p.Gln752Pro) | VUS | Variation ID: 959252 | 1 |
| *BARD1* | c.2279C>A (p.Ser760*) | VUS | Variation ID: 934257 | 1 |
| *BLM* | c.191A>T (p.Asp64Val) | VUS | rs140382474 | 1 |
| *BLM* | c.543C>A (p.Ser181Arg) | VUS | rs761288442 | 1 |
| *BLM* | c.934T>G (p.Ser312Ala) | VUS | rs372454889 | 1 |
| *BLM* | c.968A>G (p.Lys323Arg) | VUS | rs146504061 | 2 |
| *BMPR1A* | c.1388G>A (p.Ser463Asn) | VUS | Variation ID: 854431 | 1 |
| *BMPR1A* | c.1433G>A (p. Arg478His) | VUS | rs113849804 | 2 |
| *BRIP1* | c.588C>G (p.Asn196Lys) | VUS | rs758851721 | 1 |
| *BRIP1* | c.2220G>T (p.Gln740His) | VUS | rs45589637 | 2 |
| *BRIP1* | c.3262C>T (p.His1088Tyr) | VUS | rs878855154 | 1 |
| *BRIP1* | c.3478A>G (p.Asn1160Asp) | VUS | Variation ID: 843942 | 1 |
| *BRIP1* | c.3651G>T (p.Trp1217Cys) | VUS | rs542698396 | 1 |
| *BRCA1* | c.1881C>G (p.Val627=) | VUS | rs80356838 | 1 |
| *BRCA1* | c.2299A>G (p.Ser767Gly) | VUS | rs80357194 | 1 |
| *BRCA1* | c.4344C>T (p.Ser1448=) | VUS | rs1250691798 | 1 |
| *BRCA2* | c.7A>G (p.Ile3Val) | VUS | rs770479195 | 1 |
| *BRCA2* | c.229A>G (p.Thy77Ala) | VUS | rs80358500 | 1 |
| *BRCA2* | c.383A>G (p.Asp128Gly) | VUS | rs80358627 | 1 |
| *BRCA2* | c.561G>C (p.Glu187Asp) | VUS | rs754678843 | 1 |
| *BRCA2* | c.2803G>C (p.Asp935His) | VUS | rs28897716 | 1 |
| *BRCA2* | c.6586A>G (P.Lys2196Glu) | VUS | rs398122561 | 1 |
| *BRCA2* | c.7559G>C (p.Arg2520Pro) | VUS | rs80358982 | 1 |
| *BRCA2* | c.7912T>C (p.Phe2638Leu) | VUS | rs876658206 | 1 |
| *BRCA2* | c.8035G>T (p.Asp2679Tyr) | VUS | rs80359040 | 1 |
| *BRCA2* | c.9078G>C (p.Gln3026His) | VUS | rs1359207910 | 1 |
| *BRCA2* | c.9628G>A (p.Gly3210Ser) | VUS | rs1064794491 | 1 |
| *CASR* | c.944C>A (p.Gly315Asp) | VUS | rs1389231880 | 1 |
| *CASR* | c.2671C>T (p.Arg891Cys) | VUS | rs1360294910 | 1 |
| *CEP57* | c.545T>A (p.Leu182His) | VUS | rs150399616 | 1 |
| *CDH1* | c.2369C>T (p.Thr790Ile) | VUS | rs587780120 | 1 |
| *CDKN1C* | c.512C>T (p.Ala171Val) | VUS | Not reported | 1 |
| *CDKN2A* | c.9_32del (p.Ala4_Pro11del) | VUS | rs587780668 | 1 |
| *CHEK2* | c.846+4_846+7del | VUS | rs764884641 | 1 |
| *CHEK2* | c.199T>G (p.Ser67Ala) | VUS | rs1601852238 | 1 |
| *CHEK2* | c.1427C>T (p.Thr476Met) | VUS | rs142763740 | 1 |
| *CTNNA1* | c.286G>C (p.Asp96His) | VUS | rs1451287719 | 1 |
| *DICER1* | c.4616C>T (p.Thr1539Met) | VUS | rs747901058 | 1 |
| *DICER1* | c.4819C>T (p.Arg1607Trp) | VUS | rs189119295 | 1 |
| *DICER1* | c.4961A>T (p.Asp1654Val) | VUS | rs1595338147 | 1 |
| *DIS3L2* | c.849G>A (p.Lys283=) | VUS | rs376763206 | 1 |
| *DIS3L2* | c.1447C>G (p.Arg483Gly) | VUS | rs186865544 | 2 |
| *DIS3L2* | c.1970G>A (p.Arg657His) | VUS | rs757443947 | 1 |
| *DIS3L2* | c.2233G>A (p.Ala745Thr) | VUS | rs758837060 | 1 |
| *ERCC3* | c.1150A>T (p.Ile384Phe) | VUS | rs1024820965 | 1 |
| *ERCC3* | c.2080G>A (p.Ala694Thr) | VUS | rs151216904 | 1 |
| *ERCC5* | c.2891G>A (p.Arg964Gln) | VUS | rs149859074 | 1 |
| *EXT1* | c.518A>G (p.Asn173Ser) | VUS | Variation ID: 910036 | 1 |
| *FAN1* | c.1129C>T (p.Arg377Trp) | VUS | rs151322829 | 1 |
| *FANCA* | c.1874G>C (p.Cys625Ser) | VUS | rs139235751 | 1 |
| *FANCF* | c.647G>C (p.Arg216Pro) | VUS | rs192534185 | 1 |
| *FH* | c.34C>T (p.Arg12Cys) | VUS | rs199912971 | 1 |
| *FH* | c.759A>G (p.Gln253=) | VUS | rs147518314 | 1 |
| *FLCN* | c.66G>A (p.Thr22=) | VUS | rs747210367 | 1 |
| *FLCN* | c.249+2C>T (splice donor) | VUS | rs939223011 | 1 |
| *FLCN* | c.303G>C (p.Glu101Asp) | VUS | rs910566279 | 1 |
| *GATA2* | c.380A>G (p.His127Arg) | VUS | rs1576749074 | 1 |
| *GATA2* | c.661A>C (p.Met221Leu) | VUS | rs1252930387 | 1 |
| *GATA2* | c.1348G>A (p.Gly450Arg) | VUS | rs370164300 | 1 |
| *GATA2* | c.1415C>T (p.Pro472Leu) | VUS | rs779338723 | 1 |
| *HNF1A* | c.92G>A (p.Gly31Asp) | VUS | rs137853247 | 1 |
| *KIT* | c.67+3G>A (Intronic) | VUS | rs1560366820 | 1 |
| *KIT* | c.200C>G (p.Thr67Ser) | VUS | rs144933028 | 1 |
| *KIT* | c.957A>G (p.Ile319Met) | VUS | rs377102206 | 1 |
| *KIT* | c.1207A>G (p.Ile403Val) | VUS | rs878853759 | 1 |
| *KMT2D* | c.1759G>A (p.Glu587Lys) | VUS | rs199980307 | 1 |
| *KMT2D* | c.6416C>T (p.Ala2139Val) | VUS | rs754730634 | 1 |
| *MAX* | c.37-5T>C | VUS | rs774765085 | 1 |
| *MAX* | c.414G>A (p.Ser138=) | VUS | rs559090673 | 1 |
| *MC1R* | c.100C>T (p.Arg34Trp) | VUS | rs376679503 | 1 |
| *MC1R* | c.464T>C (p.Ile155Thr) | VUS | rs1110400 | 1 |
| *MEN1* | c.236C>G (p.Pro79Arg) | VUS | rs1555166557 | 1 |
| *MET* | c.50C>T (p.Thr17Ile) | VUS | rs747777018 | 1 |
| *MET* | c.362T>C (p.Val121Ala) | VUS | rs879254339 | 1 |
| *MET* | c.1232C>T (p.Ala411Val) | VUS | rs201154533 | 1 |
| *MET* | c.1640G>A (p.Arg547Gln) | VUS | rs761951444 | 1 |
| *MET* | c.2086del (p.Thr696Hisfs*4) | VUS | Variation ID: 959253 | 1 |
| *MET* | c.2486T>A (p.Leu829Gln) | VUS | rs1554396554 | 1 |
| *MET* | c.2622T>G (p.Asn874Lys) | VUS | rs118057172 | 1 |
| *MLH3* | c.1925T>C (p.Phe642Ser) | VUS | rs777245092 | 1 |
| *MLH3* | c.2911G>A (p.Val971Ile) | VUS | rs41555714 | 1 |
| *MSH2* | c.1130A>G (p.Gln377Arg) | VUS | rs776174711 | 1 |
| *MSH2* | c.1748A>T (p.Asn583Ile) | VUS | rs201118107 | 1 |
| *MSH2* | c.1254A>G (p.Ile418Met) | VUS | rs751431238 | 2 |
| *MSH2* | c.2647A>G (p.Ile883Val) | VUS | Not reported | 1 |
| *MSH3* | c.1366G>A (p.Glu456Lys) | VUS | rs963234468 | 1 |
| *MSH3* | c.2016T>G (p.Ile672Met) | VUS | rs139593361 | 1 |
| *MSH3* | c.2917A>G (p.Ile973Val) | VUS | rs762608251 | 1 |
| *MSH6* | c.818G>T (p.Gly273Val) | VUS | rs769610487 | 1 |
| *MSH6* | c.1730G>A (p.Arg577His) | VUS | rs376220212 | 1 |
| *MSH6* | c.3198T>C (p.Tyr1066=) | VUS | rs199643502 | 1 |
| *MUTYH* | c.125A>G (p.Asn42Ser) | VUS | rs563275223 | 1 |
| *MUTYH* | c.1309C>T (p.Arg437Trp) | VUS | rs587778540 | 1 |
| *NBN* | c.119C>T (p.Ser40Leu) | VUS | rs587781530 | 1 |
| *NBN* | c.266G>C (p.Arg89Pro) | VUS | rs747315554 | 1 |
| *NBN* | c.613A>G (p.Ile205Val) | VUS | rs730881845 | 1 |
| *NBN* | c.1454C>T (p.Thr485Met) | VUS | rs200891292 | 1 |
| *NBN* | c.2009G>A (p.Arg670Lys) | VUS | rs1554556522 | 1 |
| *NF1* | c.7457C>T (p.Thr2486Ile) | VUS | rs149055633 | 1 |
| *NF2* | c.243_248del (p.Leu82_Asp83del) | VUS | rs777858863 | 1 |
| *NF2* | c.952G>A (p.Val318Ile) | VUS | rs996057882 | 1 |
| *NTHL1* | c.49C>G (p.Leu17Val) | VUS | Not reported | 1 |
| *NTHL1* | c.65G>A (p.Ser22Asn) | VUS | rs377360166 | 1 |
| *PALB2* | c.656A>G (p.Asp219Gly) | VUS | rs45594034 | 1 |
| *PALB2* | c.1588C>T (p.Leu530Phe) | VUS | rs876659671 | 1 |
| *PALB2* | c.2453T>C (p.Phe818Ser) | VUS | rs370908330 | 1 |
| *PALB2* | c.2834+4T>C | VUS | rs982643702 | 1 |
| *PALB2* | c.3412G>A (p.Ala1138Thr) | VUS | rs577651839 | 1 |
| *PALB2* | c.3539T>C (p.Ile1180Thr) | VUS | rs180177139 | 1 |
| *PDFRA* | c.1147C>T (p.Arg383Cys) | VUS | rs1324781921 | 1 |
| *PDGFRA* | c.1338G>T (p.Glu446Asp) | VUS | Variation ID: 838620 | 1 |
| *PMS1* | c.799G>A (p.Val267Ile) | VUS | rs947219738 | 1 |
| *PMS2* | c.241G>A (p.Glu81Lys) | VUS | rs730881919 | 1 |
| *PMS2* | c.989-3T>C (Intronic) | VUS | rs1156325177 | 2 |
| *PMS2* | c.2350G>A (p.Asp784Asn) | VUS | rs143340522 | 1 |
| *POLD1* | c.73G>A (p.Asp25Asn) | VUS | rs1426253750 | 1 |
| *POLD1* | c.224T>C (p.Ile75Thr) | VUS | rs878854534 | 1 |
| *POLD1* | c.353C>T (p.Ser118Phe) | VUS | rs780604625 | 1 |
| *POLD1* | c.613G>A (p.Gly205Ser) | VUS | rs914238978 | 1 |
| *POLD1* | c.632G>A (p.Arg211His) | VUS | rs373192520 | 1 |
| *POLD1* | c.3073G>A (p.Val1025Met) | VUS | rs1060501858 | 1 |
| *POLD1* | Gain (Exons 2-19) | VUS | Not reported | 1 |
| *POLE* | c.2276G>A (p.Arg759His) | VUS | rs746774432 | 1 |
| *POLE* | c.2465_2467dupAGG | VUS | rs1237046519 | 1 |
| *POLE* | c.2773T>C (p.Ser925Pro) | VUS | rs141552148 | 1 |
| *POLE* | c.3721_3722delinsTC (p.Glu1241Ser) | VUS | Variation ID:  405860 | 1 |
| *POLE* | c.577A>G (p.Ser193Gly) | VUS | rs760588718 | 1 |
| *POLE* | c.6445C>T (p.Arg2149Cys) | VUS | rs771490182 | 1 |
| *POLE* | c.6775C>T (p.Arg2259Trp) | VUS | rs866548835 | 1 |
| *PTCH1* | c.1286A>G (p.Asp429Gly) | VUS | rs1588602719 | 1 |
| *PTCH1* | c.1760T>C (p.Met587Thr) | VUS | rs1588578473 | 1 |
| *PTCH1* | c.2215_2216delCAinsTT (p.His739Phe) | VUS | rs864622295 | 1 |
| *RAD50* | c.55G>T (p.Asp19Tyr) | VUS | rs770566177 | 1 |
| *RAD50* | c.1663A>G (p.Ile555Val) | VUS | rs201120953 | 1 |
| *RAD50* | c.3559G>T (p.Val1187Leu) | VUS | rs771855501 | 1 |
| *RAD51C* | c.571+4A>G | VUS | rs587780257 | 1 |
| *RAD51D* | c.26G>C (p.Cys9Ser) | VUS | rs140825795 | 2 |
| *RAD51D* | c.86T>C (p.Val29Ala) | VUS | rs1567735899 | 1 |
| *RAD51D* | c.715C>T (p.Arg239Trp) | VUS | rs770250516 | 1 |
| *RB1* | c.2464C>G (p.Pro822Ala) | VUS | rs368413787 | 1 |
| *RB1* | c.2557T>C (p.Cys853Arg) | VUS | rs1295589257 | 1 |
| *RECQL4* | c.2671C>A (p.Pro891Thr) | VUS | rs757175211 | 1 |
| *RECQL4* | c.1199A>C p.(Lys400Thr) | VUS | rs1489838661 | 1 |
| *RECQL4* | c.3055+5G>A | VUS | rs377031190 | 2 |
| *RECQL4* | c.3268G>A (p.Glu1090Lys) | VUS | rs756401653 | 1 |
| *RECQL4* | c.3545_3546delinsTT (p.Arg1182Leu) | VUS | Variation ID:  856454 | 1 |
| *RECQL4* | c.3622C>G (p.Arg1208Gly) | VUS | rs41555416 | 1 |
| *RET* | c.785T>C (p.Val262Ala) | VUS | rs139790943 | 1 |
| *RET* | c.2166G>T (p.Lys722Asn) | VUS | rs527726480 | 1 |
| *RET* | c.3056C>T (p.Ala1019Val) | VUS | rs1371891301 | 1 |
| *SDHA* | c.260C>T (p.Thr87Ile) | VUS | rs756543943 | 1 |
| *SDHA* | c.290G>C (p.Arg97Thr) | VUS | rs371274523 | 1 |
| *SDHA* | c.955A>C (p.Ile319Leu) | VUS | rs377509915 | 1 |
| *SDHA* | c.1919A>G (p.Glu640Gly) | VUS | rs372480044 | 1 |
| *SDHAF2* | c.59G>A (p.Ser20Asn) | VUS | Not Reported | 1 |
| *SDHB* | c.307A>G (p.Met103Val) | VUS | rs140178341 | 1 |
| *SLX4* | c.467C>A (p.Thr156Lys) | VUS | rs144614070 | 1 |
| *SLX4* | c.3607C>G (p.Pro1203Ala) | VUS | rs745508761 | 1 |
| *SMARCA4* | c.2110C>T (p.Arg704Trp) | VUS | rs781539973 | 1 |
| *SMARCA4* | c.3249G>C (p.Glu1083Asp) | VUS | rs1600335341 | 1 |
| *SMARCE1* | c.1103A>G (p.Glu368Gly) | VUS | rs1555605086 | 1 |
| *STK11* | c.877G>A (p.Glu293Lys) | VUS | rs398123405 | 1 |
| *STK11* | c.952G>A (p.Glu318Lys) | VUS | rs587781966 | 1 |
| *SUFU* | c.1060G>A (p.Ala354Thr) | VUS | rs1554854570 | 1 |
| *SUFU* | c.1379A>G (p.Lys460Arg) | VUS | rs778125780 | 1 |
| *TERC* | n.295A>G (RNA change) | VUS | Variation ID: 856455 | 1 |
| *TERT* | c.2368G>A (p.Val790Ile) | VUS | rs371413388 | 1 |
| *TERT* | c.3268G>A (p.Val1090Met) | VUS | rs121918664 | 1 |
| *TMEM127* | c.343G>A (p.Val115Ile) | VUS | rs930453941 | 1 |
| *TMEM127* | c.407C>T (p.Thr136Met) | VUS | rs759138897 | 1 |
| *TSC2* | c.98G>A (p.Gly33Asp) | VUS | rs1352220583 | 1 |
| *TSC2* | c.644T>C (p.Ile215Thr) | VUS | rs1555498571 | 1 |
| *TYR* | c.290G>A (p.Gly97Glu) | VUS | Not reported | 1 |
| *TP53* | c.37C>T (p.Pro13Ser) | VUS | rs1060501208 | 1 |
| *TP53* | c.560-9T>G | VUS | rs794727781 | 1 |
| *TP53* | c.941C>G (p.Ser314Cys) | VUS | Variation ID: 848532 | 1 |
| *WRN* | c.674G>A (p.Arg225Gln) | VUS | rs62506077 | 1 |
| *WRN* | c.1027G>A (p.Glu343Lys) | VUS | rs11574222 | 1 |
| *WRN* | c.3070A>G (p.Thr1024Ala) | VUS | rs1563376455 | 1 |
| *XPC* | c.2404G>A (p.Gly802Ser) | VUS | rs200148127 | 1 |

Abbreviations: VUS, Variants of uncertain significance.
