# Supplementary material for: Germline molecular data in hereditary breast cancer in Brazil: Lessons from a large single-center analysis
Source: PLoS One. 2021 Feb 19;16(2):e0247363. doi: 10.1371/journal.pone.0247363 (PMC7895369; doi:10.1371/journal.pone.0247363)
Supplement: Attachment — Submitted filename: Rebuttal letter-2nd revision.docx [file pone.0247363.s006.docx]

**Manuscript PONE-D-20-32343**

**Response to the academic editor and reviewers**

Dear Dr. Toland,

Thank you for giving us the opportunity to submit a second revised version of the manuscript “Germline molecular data in hereditary cancer in Brazil: lessons from a large single-center analysis”. We are grateful for the time and effort that you and the reviewers dedicated to providing feedback on our manuscript. All the comments provided the possibility of relevant improvements to our manuscript.

We have incorporated the suggestions made by the reviewer1. Authors´ responses are described in **blue**. Changes performed in the revised manuscript are highlighted in yellow. Page numbers refer to the revised manuscript file with tracked changes.

Reviewer #1:

1. One point that needs to be repaired is the sentence in page 3: “Even though most of the published data on hereditary BC among Latin American countries are from Brazil [11]” referring to the reference 11, should be rephrased since it might induce to a wrong concept that much of the contribution in Latin America is by the number of publications from Brazil, which is wrong. That review it is associated to PARP inhibitors and immunotherapy (stated in the title) which, besides, it is not reflecting the sense of this sentence. More important in that review, among most of the papers from Brazil, few ones refer to genes’ panels (a concept immediately expressed in the MS), many of the referenced Brazilian papers are biased by methodology limitations or patients´ selection, many refers to TP53 solely and, to be rescued, two references reflect the meaning of the sentence (ref 42 and ref 60). Importantly, published works in Latin America referred in the same review reflects the experience in a large series of cases that no one has reached at the year of publication (ref 98 and 100), these four publications are the ones that deserve a comment if that sentence is kept.

**Author response: We fully agree and apologize for this misunderstanding. This comment was very important. We rephrased the sentence and included two references.**

**Changes in the text of the revised manuscript: page 3 line 40-41, “**Even though there are published data on hereditary BC among Latin American countries [11,12], few studies have included non-*BRCA* genes and multigene panel testing [13,14]. Brazil is the largest country in South America and is the most genetically heterogeneous [14]. Most studies performed in Brazilian cohorts on hereditary breast cancer have been performed in the Southern and Southeastern parts of the country [12,15]. For this reason, epidemiological data about the prevalence of cancer predisposition syndromes in other parts of the country are needed.”

2. A reference should be added in the sentence pg. 26 line 263: “Our results are in concordance with previous reports stating that 13% of patients with multiple primary BC harbor PVs in BRCA2, CHEK2, BARD1, and RAD51C

**Author response: Thank you very much for this comment. We added this missing information and rephrased the data to better express our results in comparison to the literature. In our cohort 13/224 (5.8%) of patients had bilateral breast cancer (BC), seven patients did not harbor pathogenic variants (PVs). Six patients harbored PVs (6/13, 46%). Four out of thirteen (31%) patients harbored a PV in non-*BRCA* genes. Shin et al. (2020) described a cohort (n=496) with 15% of bilateral BC, 31,6% harbored PVs in non-*BRCA* genes (*CHEK2, TP53, NBN, CDH1, MRE11A*). Fanale et al. (2020) suggested that all bilateral BC patients should be offered multigene testing. They analysed a cohort of 139 cases of bilateral BC, 35.8% (19/53) harbored PVs in non-*BRCA* genes (*PTEN, PALB2, CHEK2, ATM, RAD51C*).**

**Changes in the text of the revised manuscript: page 15 line 262-266, “**Patients with cancer predisposition syndromes have a higher risk of developing a second primary BC, especially for *BRCA 1/2* mutation carriers [45]. Nevertheless, 8 - 36% of patients with bilateral BC harbor PVs in other genes beyond *BRCA* [32,47,48]. Bilateral BC represented 5.8% (13/224) of our cohort. Thirty-one percent (4/13) of patients with bilateral BC harbored PVs in non-*BRCA* genes (*CHEK2*, *BARD1* and *RAD51C*). These results are in concordance with previous studies [32,47].”
